# 3D point cloud lithology identification based on stratigraphically constrained continuous clustering

**DOI:** 10.1038/s41598-025-18946-3

**Published:** 2025-10-07

**Authors:** Binqing Gan, Ran Jing, Yanlin Shao, Yuangang Liu, Xiaolei Duan, Peijin Li, Longfan Li

**Affiliations:** School of Geosciences, Yangtze University, Wuhan, 430100 China

**Keywords:** Geological outcrop, Lithology identification, Point cloud segmentation, Machine learning, Environmental sciences, Natural hazards, Solid Earth sciences

## Abstract

Three-dimensional laser scanning provides high-precision spatial data for automated lithology identification in geological outcrops. However, existing methods exhibit limited performance in transition zones with blurred boundaries and demonstrate reduced classification accuracy under complex stratigraphic conditions. This study proposes a Stratigraphically Constrained Continuous Clustering (SCCC) framework to address these limitations. The framework incorporates sedimentological principles of lateral continuity through a dynamic density-threshold hierarchical clustering algorithm that optimizes lithological unit boundaries using adjacency-based cluster merging criteria. A patch-level feature aggregation module, integrated within the proposed SCCC framework, constructs a multimodal feature space by aggregating geometric covariance matrices and spectral distribution entropy into compact patch-level feature vectors. Random forest classifier subsequently performs lithology discrimination. Experimental validation using the Qingshuihe Formation outcrop dataset demonstrates that SCCC achieves overall accuracy of 94.64%, F1-score of 94.58%, and mean intersection over union of 90.87%. These results surpass traditional machine learning (SVM, XGBoost) and deep learning methods (PointNet) by 26.22–68.36%, indicating substantial improvements in classification accuracy and boundary delineation within transition zones. SCCC particularly enhances recognition capabilities for sandstone-mudstone thin interbeds and conglomerate-sandstone transitional zones. Ablation experiments confirm that stratigraphic constraints effectively suppress noise while improving computational efficiency, reducing memory usage by 83.3% and processing time by 85.7%. This method provides a high-precision, interpretable technical pathway for intelligent geological exploration through deep integration of geological principles with computational models.

## Introduction

The rapid development of remote sensing and computer vision technologies has established^1^ 3D laser scanning as an indispensable tool in geological exploration^2–4^. As a high-precision spatial data carrier, 3D point clouds effectively characterize critical geological elements, including outcrops, stratigraphic interfaces, and fault structures. However, existing methods still face significant challenges in classification accuracy when applied to complex terrains and heterogeneous lithologic assemblages, representing a major bottleneck in advancing intelligent geological exploration.

Lithology identification has evolved from 2D to 3D techniques. Early studies relied on optical image analysis^5,6^ where manual interpretation of core samples or outcrop textures and colors dominated. Such 2D frameworks, constrained by projection distortions and limited feature dimensions, struggled to meet the demands of complex geological scenarios. 3D laser scanning technology, by capturing millimeter-level spatial coordinates, reflectance intensity, and multimodal information^7–9^provides a more comprehensive basis for lithology discrimination. Handcrafted geometric features (e.g., curvature, density distribution) combined with traditional machine learning^10–13^ algorithms like support vector machines (SVM)^14,15^ have improved recognition accuracy. Despite these advancements, machine learning methods remain limited by the representational capacity of manual features. In contrast, deep learning^16^ enables automatic high-dimensional feature extraction through end-to-end learning, uncovering nonlinear relationships within outcrop point clouds. Notably, architectures such as PointNet^17–19^ and its variants (e.g., PointNet + + ^20–22^, PointCNN^23–25^ directly process unstructured point clouds, accelerating their application in geological analysis. Nevertheless, current lithology identification research faces two critical issues: (1) Most methods adopt a holistic classification strategy, failing to accurately delineate lithological unit boundaries, leading to confusion in transitional zones, (2) Overreliance on geometric features neglects the integration of geological prior knowledge, such as stratigraphic continuity^26^.

To address these limitations, this study proposes a Stratigraphically Constrained Continuous Clustering (SCCC) framework for 3D point cloud lithology identification. The method incorporates sedimentological principles of lateral continuity^27–29^ and adjacency-based cluster merging criteria^30^. Key innovations include:1) a dynamic density-threshold clustering algorithm to enhance boundary precision of lithologic units, 2) a patch-level feature aggregation module integrated within the proposed SCCC framework that fuses geometric covariance matrices^31–33^ and spectral distribution entropy to improve classification robustness. Experimental validation on the Qingshuihe Formation outcrop in the Junggar Basin demonstrates the method’s superior performance in complex lithological scenarios, offering a novel technical pathway for intelligent geological exploration.

## Methodology

**Fig. 1:**
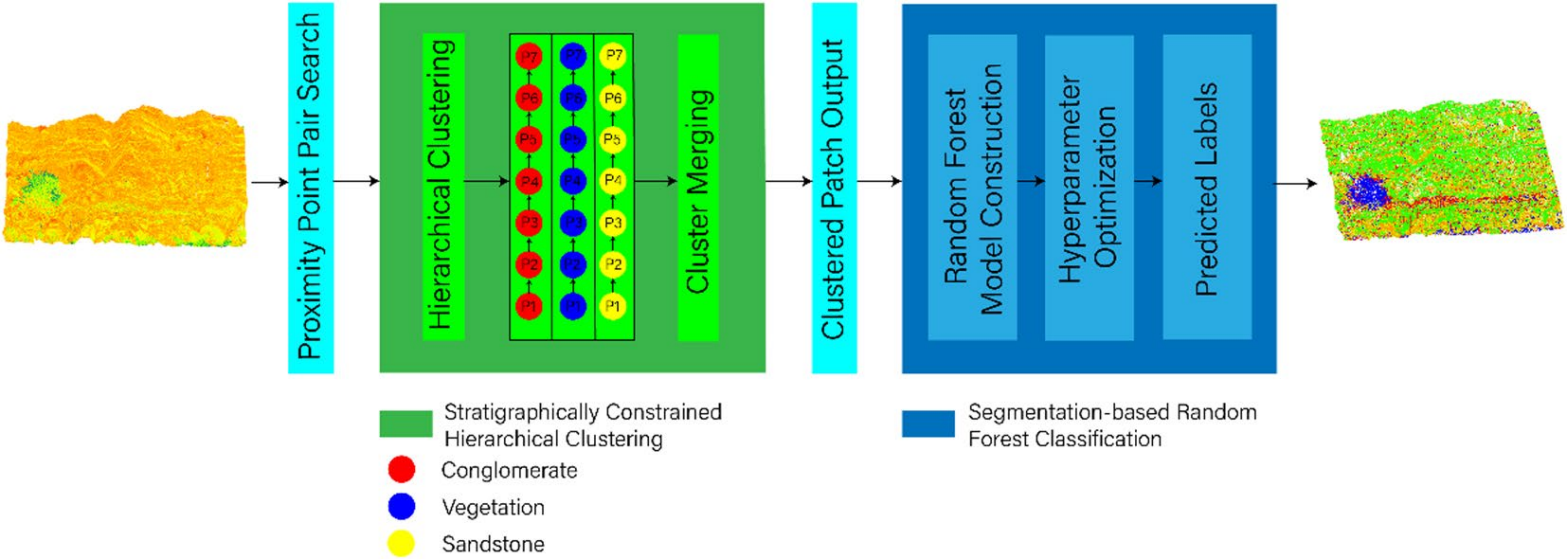
Technical workflow of 3D point cloud lithology identification based on stratigraphically constrained clustering.

The proposed framework for stratigraphically constrained lithology identification in geological outcrop point clouds comprises two main stages: (1) Hierarchical clustering based on stratigraphic inheritance laws^34^ which achieves preliminary lithological unit division through geometric and spatial distribution features, (2) Fusion feature classification of segmented objects, where a feature set incorporating geometric-morphological parameters and spectral statistics is constructed, and a random forest^35^ classifier is employed for lithology discrimination. The technical workflow is illustrated in Fig. 1.

### Experimental data

#### Study area overview

**Fig. 2:**
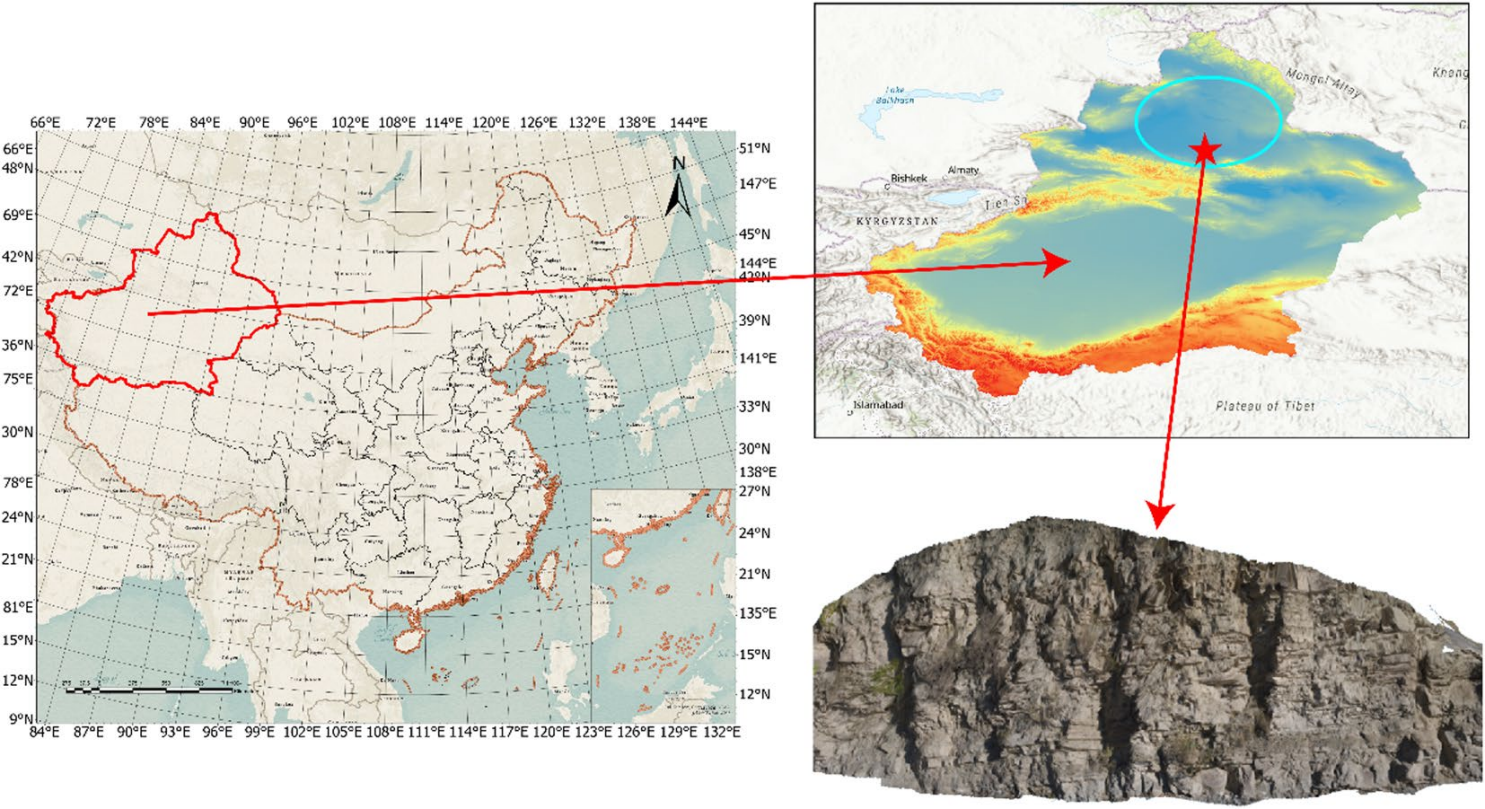
Geological Structure Distribution of the Yueyawan Outcrop in Qingshuihe Formation, Southern Junggar Basin.

The geological outcrop point cloud data used in this study were acquired from the Yueyawan outcrop of the Qingshuihe Formation in the southern margin of the Junggar Basin, Xinjiang. The lithology primarily comprises mudstone, sandstone, and conglomerate. The lower part of the Qingshuihe Formation exhibits fan-delta sedimentary environments, featuring distributary channel conglomerates, subaqueous distributary channel sandstones, mouth bar sandstones, and interchannel mudstones. The middle section transitions to a shallow lacustrine environment with mudstone-sandstone interbeds showing “mud-enveloped-sand” characteristics. The upper part gradually shifts to a semi-deep lacustrine environment dominated by horizontally bedded mudstones. The outcrop profile is well-exposed, with clear lithological variations and an overall geological structure characterized by “east-west segmentation and north-south zonation.” The dataset includes samples of siltstone, sandstone, mudstone, conglomerate, regolith, and vegetation (Fig. 2).

#### Data acquisition and preprocessing

**Fig. 3:**
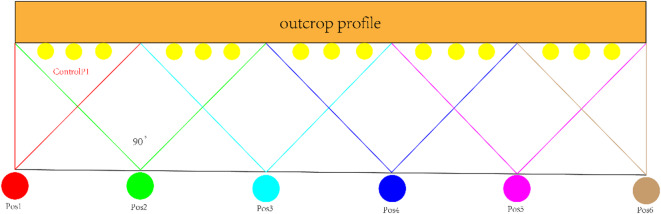
Terrestrial 3D Laser Scanning System Station Setup.

The point cloud data were acquired using the RIEGL VZ-400 terrestrial laser scanner, which operates at a near-infrared wavelength of 1550 nm. Although the device does not perform multispectral or hyperspectral sensing, the amplitude of the returned laser pulse varies with surface reflectivity in the near-infrared band. Higher reflectivity (e.g., on bare rock surfaces) produces stronger return signals, while lower reflectivity (e.g., on vegetation or weathered surfaces) yields weaker signals. These variations in pulse amplitude provide additional contrast for distinguishing lithologies. Scanner specifications, including wavelength, pulse repetition rate, and return intensity calibration, are detailed in the RIEGL VZ-400 Operating Manual^36^. Core acquisition parameters included a scanning radius of 10 m, angular resolution of 0.001 rad, and positional accuracy of ± 1 mm. The station deployment scheme is illustrated in Fig. 3, ensuring comprehensive point cloud coverage and registration accuracy. Data acquisition followed a phased scanning strategy: (1) Panoramic Pre-scanning Phase: A 360° scene reconstruction was performed at an angular resolution of 0.001 rad, (2) Key Area Enhancement Scanning: High-precision scanning was conducted for critical geological interfaces. Synchronously, high-resolution digital cameras were employed to capture orthorectified images of the outcrop, with adjacent photos ensuring a 15% overlap for subsequent stitching and visual interpretation. The detailed specifications of the datasets and scanning parameters are summarized in Table 1.

**Table 1:** Instrument Parameters​​.

Model	Pulse Frequency (kHz)	Acquisition Rate (points/s)	Scanning Speed (lines/s)	Field of View (°)	Distance Accuracy (mm/m)	Angular Resolution (°)
RIGEL VZ400	1200	500,000	< 100	360 × 100	± 5/50	< 0.001

The raw point cloud data were stored in `.txt` format and included 3D coordinates (X, Y, Z), amplitude, relative reflectance, and scanning indices (row and column). The amplitude represents the original intensity of the laser echo, while the relative reflectance is derived from the internal distance correction model of the RIEGL VZ-400 scanner. Although the device performs basic distance correction, variations in intensity caused by oblique incidence angles—especially on rugged or steep lithologic surfaces—require additional compensation. To address this, a manual incidence angle correction was performed using RiSCAN PRO 2.9 (RIEGL Laser Measurement Systems GmbH). Specifically, the “Incidence Angle Normalization” function within the Reflectance Calibration module was enabled to adjust return intensities based on the scan geometry and laser beam orientation, following the procedures described in the RiSCAN PRO user manual. This step improves radiometric consistency across the point cloud and minimizes reflectance distortion in transition zones.

**Fig. 4:**
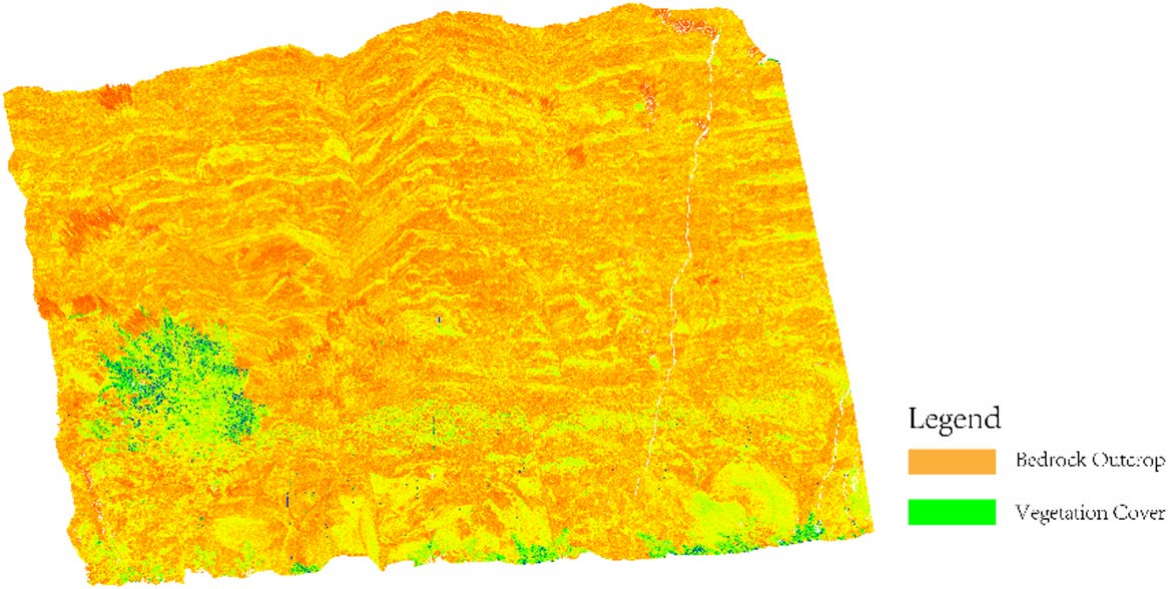
3D Topographic Features and Spatial Lithology Distribution (Yellow/Orange: Bedrock Exposure; Green: Vegetation Coverage).

Subsequent preprocessing steps—including multi-station registration, noise filtering, and statistical outlier removal—were also conducted within RiSCAN PRO. After preprocessing, the refined dataset comprised 56,063,397 valid points, as illustrated in Fig. 4.

The color gradient in Fig. 4 reflects the spatial density distribution of the point cloud. Yellow and orange gradients indicate high-density sandstone/conglomerate and medium-density mudstone/siltstone units, respectively, while green regions represent low-density vegetation-covered areas. The preprocessed data preserve the stratigraphic sequence characteristics of the Qingshuihe Formation, with clear spatial distribution patterns of sandstone-mudstone interbedded structures and conglomerate sedimentary bodies, fulfilling the requirements for lithology identification.

### Segmentation of geological outcrop point clouds based on stratigraphic continuity

In sedimentology, stratigraphic units obey two fundamental principles: Steno’s Law of Original Horizontality and the Principle of Lateral Continuity. These imply that strata of the same age are laterally continuous and maintain internal lithological homogeneity, unless truncated by later tectonic or erosional processes. Such stratigraphic inheritance provides a natural constraint that can be encoded into computational models of geological outcrop segmentation. Most existing point cloud segmentation methods rely purely on geometric or statistical criteria (e.g., density-based clustering), which often leads to fragmented boundaries in transitional zones. By embedding stratigraphic inheritance rules (e.g., dip consistency, lateral continuity, hierarchical layering) into the clustering algorithm, the proposed SCCC framework explicitly encodes geological prior knowledge, ensuring that the resulting patches are not only geometrically coherent but also geologically plausible. This stratigraphically constrained approach represents a methodological advance over unconstrained machine learning methods.

**Fig. 5:**
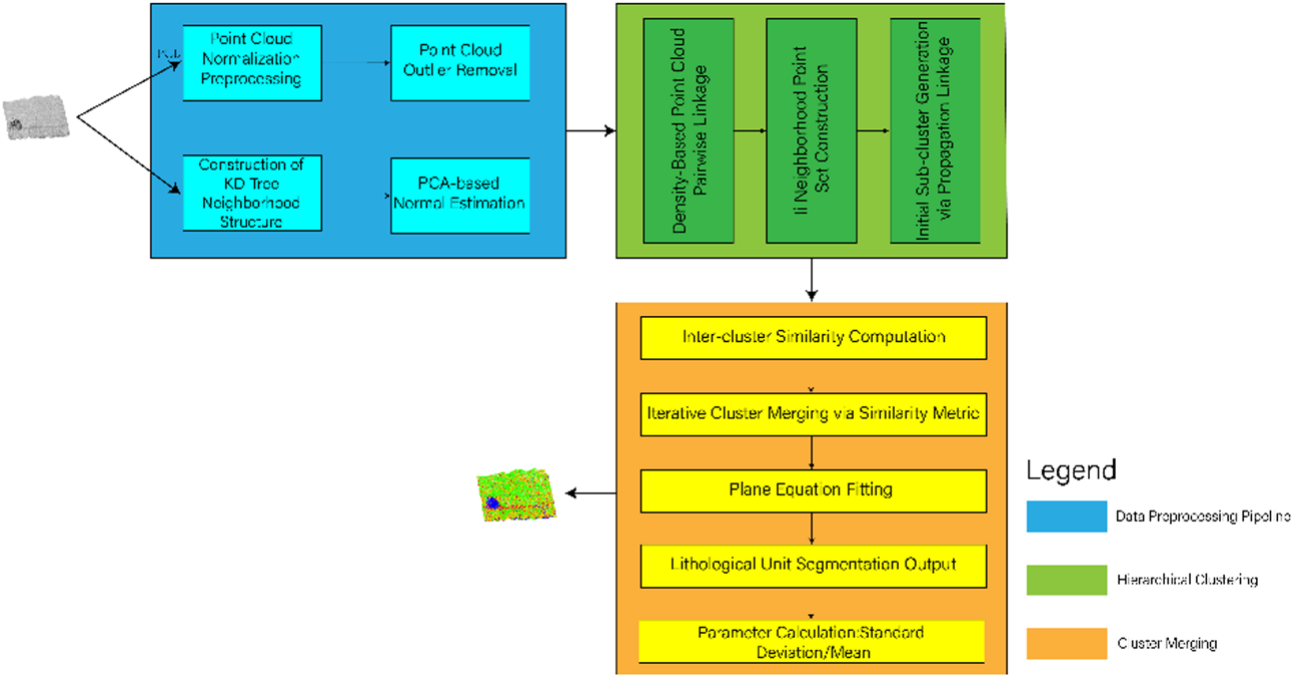
Technical flowchart of 3D point cloud segmentation based on hierarchical clustering. Data Preprocessing (Blue Modules): Outlier removal via RANSAC algorithm. Point cloud normal estimation using principal component analysis (PCA). Hierarchical Clustering (Green Modules): Dynamic density-threshold hierarchical clustering algorithm constrained by sedimentological lateral continuity principles.

Effective segmentation of geological outcrop point clouds is a critical factor influencing lithology identification accuracy. The core challenge lies in integrating geometric and spatial distribution features of point clouds with prior knowledge of stratigraphic continuity to achieve lithological unit partitioning under stratigraphic sequence constraints. To address this, this study proposes a stratigraphic inheritance-guided clustering framework for geological outcrop segmentation, as illustrated in Fig. 5.

Neighborhood search using cutoff distance d_c_ to partition initial stratigraphic units. Cluster Merging (Orange Modules): Iterative merging of fragmented patches in transition zones based on adjacency density continuity criteria (see Eq. (4)). Boundary optimization through stratigraphic dip consistency checks and spatial topological constraints.

#### Stratigraphically constrained continuous Clustering — Hierarchical segmentation

Traditional clustering methods, such as DBSCAN or octree-based partitioning, typically rely solely on point density or geometric proximity, often producing discontinuous patches in transitional regions. In contrast, the proposed SCCC algorithm embeds stratigraphic constraints directly into the clustering process. The continuity of bedding orientation, lateral extension of sedimentary units, and dip consistency are used as additional decision criteria for merging clusters. This integration ensures that the resulting patches are not only mathematically well-defined but also consistent with geological processes of sediment deposition.

**Fig. 6:**
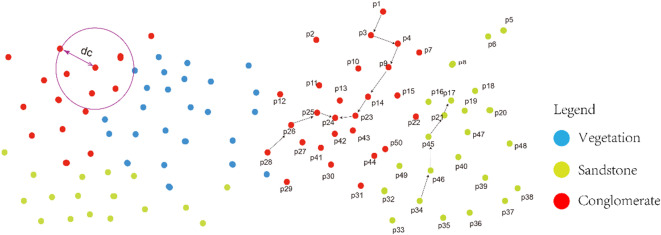
Evolution of hierarchical clustering centers and neighborhood truncation analysis (P24, P17: cluster centers; →: chain merge path; purple circles: neighborhood sets under cutoff distance d_c_).

Discrete point cloud segmentation via block aggregation significantly enhances the integrity and accuracy of lithology identification. The principle of stratigraphic continuity provides critical constraints for point cloud segmentation. According to the Law of Original Horizontality (Steno’s Law)^37^ and the Principle of Lateral Continuity^38^a single sedimentary unit maintains lithological homogeneity in its lateral distribution. Building on these foundational geological principles, a stratigraphic inheritance-guided hierarchical clustering algorithm is developed. The technical workflow comprises three key steps: (1) Data Cleaning: A Random Sample Consensus (RANSAC) algorithm is applied to fit local structural planes. Outliers deviating beyond 3σ from the structural plane are removed to mitigate interference from weathered surfaces. (2) Normal Vector Extraction: Local surface normal are computed using principal component analysis (PCA). A neighborhood radius *r* = 5 the average point spacing is set to construct covariance matrices and extract normal vector features. (3) Density Peaks Clustering: Under stratigraphic continuity constraints, initial outcrop point cloud clusters are partitioned based on geometric similarity of neighboring points. The clustering process propagates along paths satisfying stratigraphic dip consistency (Fig. 6).

In the figure, for the point pair p1→p3, since p3 and p1​ belong to the same category and are nearest neighbors, p3​ is updated as the cluster center, and clustering is performed using p3​. Through calculation, the local density of the p3→p4 pair is maximized, establishing p3 as the cluster center. Similarly, propagation paths (p1, p3), (p3, p4), (p4, p9), (p9, p14), (p14, p23), and (p23​, p24​) satisfy the stratigraphic dip consistency check. Consequently, hierarchical clustering proceeds along the sequence p1→p3→p4→p9→p14→p23→p24.

To balance over-segmentation and under-segmentation effects, a user-defined scale parameter was introduced into the neighborhood density computation stage of the hierarchical clustering framework. This parameter controls the cutoff distance d_c_ during local region search and directly affects the granularity of resulting stratigraphic units. To assess its influence on classification performance, a scale sensitivity analysis was later conducted (see Sect. 4.2).

For each point pi​, the distance to its nearest neighbor is recorded as d_cn_​. The cutoff distance d_c​_ is calculated using the formula:

1 $$\:\begin{array}{c}{\text{d}}_{\text{c}}=scale\times\:median\left({\text{d}}_{\text{c}\text{n}}\right)\end{array}$$

where scale is a tunable parameter. The cutoff distance set is derived by multiplying scale with the median of d_cn_​. Points within a distance threshold d_c_​ from pi are assigned to its neighborhood set I_i_​, as illustrated by the purple neighborhood regions in Fig. 6.

#### Cluster merging

**Fig. 7:**
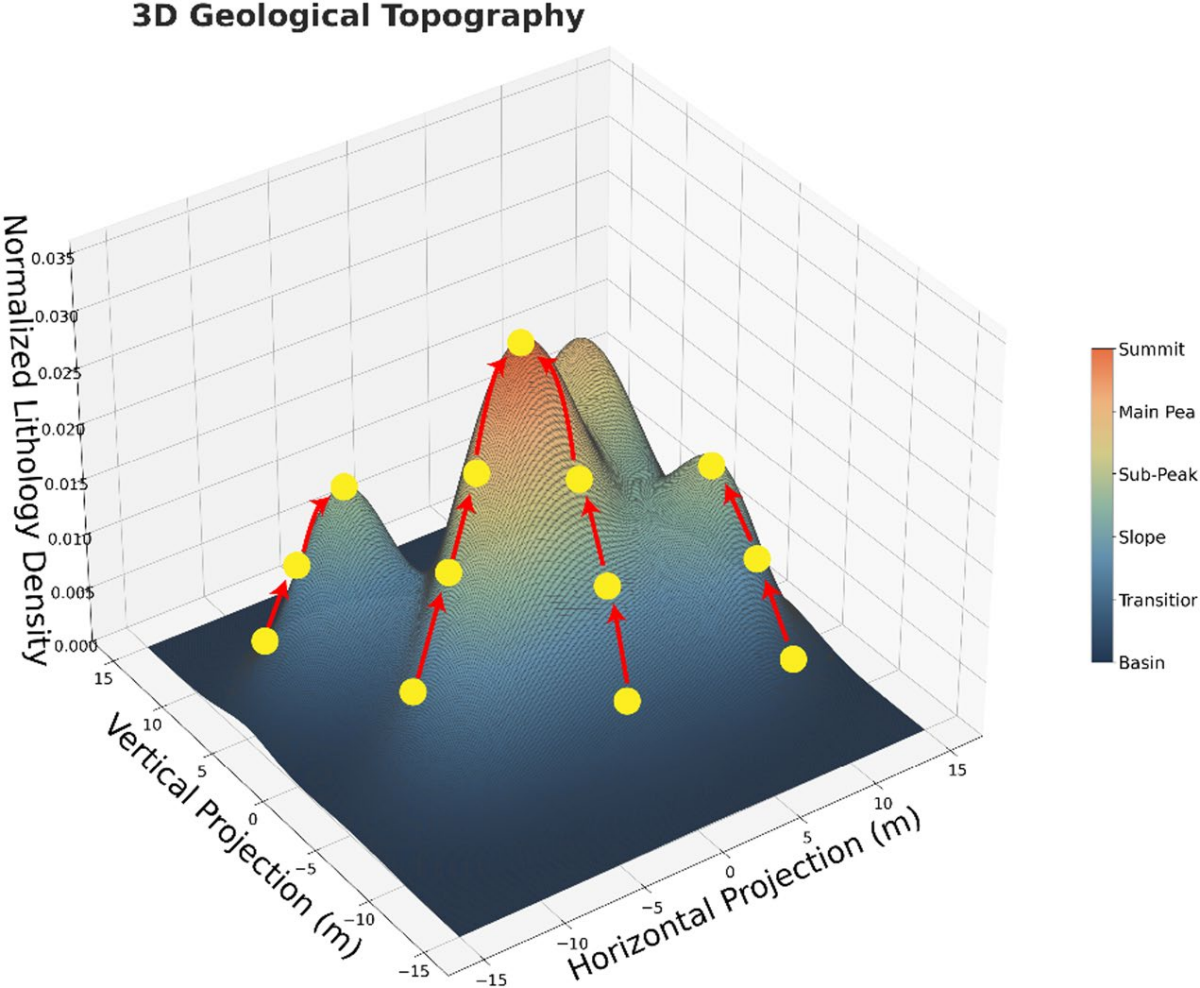
3D point cloud lithology clustering and merging (X-axis: horizontal spatial distribution/east-west orientation; Y-axis: vertical spatial distribution/north-south orientation; Z-axis: normalized lithological density index; colorbar: lithological density index).

To eliminate fragmented segmentation results in lithological transition zones and enhance boundary integrity, this study proposes a density-constrained iterative merging strategy, as illustrated in Fig. 7.

Table 2 provides a step-by-step implementation, with kernel density, boundary density, and the merge criterion referenced in Eqs. (2)– (4).

**Table 2:** Stratigraphically constrained continuous clustering (SCCC).

Step	Input/Operation	Description
1	Input	Load point cloud dataset *P* and initialize parameters: search radius *r*, stratigraphic dip angle θ, density threshold δ.
2	Neighbor search	Build k-d tree for efficient nearest-neighbor search within radius *r*.
3	Feature computation	For each point *p*_*i*_ ∈ *p*, compute local normal vector and dip direction.
4	Initial clustering	Merge points into preliminary clusters based on spatial proximity and dip consistency.
5	Stratigraphic merging	Apply hierarchical clustering to merge adjacent clusters under stratigraphic continuity constraints (lateral continuity and dip consistency).
6	Output	Generate stratigraphically constrained patches as segmentation results for subsequent lithology classification.

The initial clusters exhibit multi-peak distributions in the kernel density space (colored surfaces), with adjacent lithological u-nits forming saddle-shaped transition zones (blue regions). These patterns align with geostatistical regionalization phenomena. The merging procedure is summarized in Table 2.

First, quantify the density characteristics for each initial clustering result *C*_*p*​_, and compute the density statistics for all points within the cluster using Eq. (2). In Eq. (2), *ρ*(*p*_*i*_​) represents the kernel density estimate of point *p*_*i*​_, indicating the enrichment level of the point cluster in this category.

2 $$\:\begin{array}{c}{\mu\:}_{p}=\frac{1}{\left|{C}_{p}\right|}\sum\:_{{p}_{i}\in\:{C}_{p}}\:\rho\:\left({p}_{i}\right),{\sigma\:}_{p}=\sqrt{\frac{1}{\left|{C}_{p}\right|-1}\sum\limits_{{p}_{i}\in\:{C}_{p}}\:\left(\rho\:\right({p}_{i})-{\mu\:}_{p}{)}^{2}}\end{array}$$

For any point *p*_*i*​_∈*C*_*p*_​ in the neighborhood point set *I*_*i*_​, if *p*_*j​*_∈*I*_*i*​_ and *p*_*j*​_∈*C*_*q*_​ (*q* ≠ *p*), record *C*_*p*_​ and *C*_*q*​_ as adjacent cluster pairs, simultaneously mark *p*_*i*​_, *p*_*j*​_ as boundary-associated points. After traversing all points, construct an adjacency cluster relationship network. For each adjacent cluster pair (*C*_*p*_​, *C*_*q*_​), compute the average density of their boundary points as shown in Eq. (3).

3 $$\:\begin{array}{c}{\rho\:}_{p}=\frac{1}{{N}_{pq}}\sum\:_{{p}_{i}\in\:{B}_{pq}}\:\rho\:\left({p}_{i}\right),{\rho\:}_{q}=\frac{1}{{N}_{qp}}\sum\limits_{{p}_{j}\in\:{B}_{qp}}\:\rho\:\left({p}_{j}\right)\end{array}$$

In Eq. (3), *B*_*pq*​_ denotes the adjacent boundary point set between *C*_*p*_​ and *C*_*q*_​, with *N*_*pq*​_ representing the total number of boundary points. When the condition in Eq. (4) is satisfied, it ensures density continuity between adjacent clusters and executes cluster merging, effectively preserving the integrity of lithological unit boundaries.

4 $$\:\begin{array}{c}{\rho\:}_{p}>{\mu\:}_{q}-{\sigma\:}_{q}\text{}\text{a}\text{n}\text{d}\text{}{\rho\:}_{q}>{\mu\:}_{p}-{\sigma\:}_{p}\end{array}$$

It should be noted that the assumption of lateral continuity may not fully apply in settings characterized by strong tectonic disturbances or erosional truncations. In such cases, stratigraphic constraints may oversimplify local lithological heterogeneity. Nevertheless, in the relatively well-preserved outcrop examined in this study, stratigraphic inheritance provides a robust guiding principle for segmentation.

### Random Forest-Based lithology identification for segmented objects

#### Aggregated features of point cloud patches and sample generation

Hierarchical clustering guided by stratigraphic inheritance generates numerous segmented point cloud patches, which exhibit rich stratigraphic semantic properties and enhance lithology classification accuracy. Among classifiers, the random forest classifier is selected due to its nonlinear modeling capability for high-dimensional features, resistance to overfitting, and interpretability. However, the classifier requires input as single-sample feature vectors. To transform segmented patches into classification samples, this study proposes a feature aggregation-based patch-to-sample conversion method.

Geometric Centroid Calculation: Extract the geometric centroid (x_c_,y_c_,z_c_) of each patch:

5 $$\:\begin{array}{c}{x}_{c}=\frac{1}{n}\sum\limits_{i=1}^{n}\:{x}_{i},{y}_{c}=\frac{1}{n}\sum\limits_{i=1}^{n}\:{y}_{i},{z}_{c}=\frac{1}{n}\sum\limits_{i=1}^{n}\:{z}_{i}\end{array}$$

where n is the number of points in the patch, and (x_i_,y_i_,z_i_) are the coordinates of the i-th point.

Mean Feature Vector: Compute the average feature vector F_avg_ for all points in a patch:

6 $$\:\begin{array}{c}{F}_{\text{a}\text{v}\text{g}}={\left[\frac{1}{n}\sum\limits_{i=1}^{n}\:{f}_{1,i},\frac{1}{n}\sum\limits_{i=1}^{n}\:{f}_{2,i},\frac{1}{n}\sum\limits_{i=1}^{n}\:{f}_{3,i}\right]}^{T}\end{array}$$

where f_k, i_ represents the k-th feature value of the i-th point.

Majority Label Assignment: Assign the most frequent lithology label within the patch as its label L_major_​. The final structured classification sample includes spatial localization features, physical property statistics, target labels, and patch identifiers.

#### Random forest model construction

The input of the lithology identification model is the aggregated features of the segmented patches. To address scale differences among patches, coordinate normalization is applied using:

7 $$\:\begin{array}{c}\stackrel{\sim}{P}=\frac{P-{\mu\:}_{P}}{\text{max}\left(\parallel\:\varvec{P}{\parallel\:}_{2}\right)}\end{array}$$

where µ_P_ is the mean vector of centroid coordinates, and ∥⋅∥_2_​ denotes the Euclidean norm.

A dynamic feature selection mechanism is implemented to filter non-discriminative features (e.g., patch identifiers) based on Gini impurity reduction. During model initialization, four critical hyperparameters are optimized for lithology recognition: (1) Number of decision trees (nestimators​), (2) Maximum tree depth (max_depth), (3) Minimum samples per leaf node (min_samples_leaf), (4) Minimum samples for node splitting (min_samples_split), Stratified sampling splits the dataset into training (95%) and testing (5%) subsets, ensuring balanced class distributions. Parallel computing accelerates feature partitioning and subtree construction. Model performance is evaluated using out-of-bag (OOB) error for generalization and weighted F1-score for class-imbalanced scenarios.

#### Hyperparameter optimization procedure

The performance of the random forest lithology identification model is highly dependent on the combination of initialization hyperparameters^35,39,40^. Key parameters and their geological implications are listed in Table 3.

**Table 3:** Impact mechanisms and tuning ranges of hyperparameters for tree-based ensemble models.

Hyperparameter	Symbol	Impact Mechanism	Search Range
Number of trees	nestimators	Reduces model variance but increases redundancy	{100,500,1000}
Maximum depth	max_depth	Controls complexity; excessive depth causes overfitting	{10, 15, 20, 25, 30}
Min. leaf samples	min_samples_leaf	Enhances generalization with larger values	{1, 3, 5}
Min. split samples	min_samples_split	Reduces noise sensitivity	{2, 5, 10}

To identify the optimal hyperparameter combination, we performed a grid search across all value combinations listed in Table 3. For each configuration, the Random Forest model was trained on 95% of the aggregated patch samples and validated on the remaining 5%, using stratified sampling to preserve class balance. Model performance was assessed using out-of-bag (OOB) error estimates and the weighted F1-score. The configuration yielding the lowest OOB error and highest weighted F1-score was selected as the final parameter set. Detailed results of this grid search are presented in Sect. 3.1.

#### Postprocessing of lithology identification results

After completing lithology discrimination of the outcrop point cloud patches, the predicted labels must be inversely mapped back to the original point cloud to obtain comprehensive lithology identification results. The core of this process lies in establishing spatial consistency constraints, ensuring that all points within the same segmented patch share a uniform lithology label. Specifically, the implementation relies on the patch cluster index appended to the attribute field during the point cloud aggregation phase. This index serves as a unique identifier for constructing index associations. A dictionary MM is generated, consisting of key-value pairs in the format < Patch Index, Predicted Label>, and a hash table is employed to optimize retrieval efficiency. This approach enables rapid matching of patch labels and completes the postprocessing of label mapping for the original point cloud. The method achieves full-scene label inversion for 50 million points, reducing computational complexity from point-level to patch-level operations. The hash table-based indexing system ensures efficient integration of segmentation and classification results while preserving spatial coherence and geological plausibility.

### Experimental setup and evaluation metrics

#### Experimental environment

The experimental platform was equipped with an Intel(R) Xeon(R) Gold 5222 CPU@3.80 GHz processor and an NVIDIA RTX A6000 GPU, supported by 256 GB DDR4 RAM and a 1 TB NVMe SSD to ensure efficient processing of large-scale point cloud data. All computational tasks were performed under a hybrid architecture: the point cloud segmentation module ran on Ubuntu 22.04 LTS, while classification model training was conducted on Windows 10 Professional Edition. Detailed configurations of the software framework and algorithm implementation are presented in Table 4.

**Table 4:** Algorithm implementation framework and technical Configurations.

Module	Language	Platform	Libraries/Tools	Functionality
Stratigraphy-constrained Clustering	C + + 14	Ubuntu 22.04 LTS	PCL 1.12.1, OpenCV 4.8.0	Normal estimation, dynamic clustering, cluster merging
Random Forest Classifier	Python 3.11	Windows 10	scikit-learn 1.3.0, NumPy 1.26	Feature aggregation, hyperparameter tuning
PointNet Benchmark	Python 3.11	Windows 10	PyTorch 2.0.1, CUDA 11.8	Point feature learning, end-to-end training
Traditional ML Models	Python 3.11	Windows 10	scikit-learn 1.3.0, XGBoost	SVM/XGBoost/K-NN implementation

#### Evaluation metrics

To quantitatively assess the effectiveness of our stratigraphically constrained segmentation and classification framework, we employ three complementary metrics that each capture a different aspect of geologically meaningful performance:

(1) Overall Accuracy (OA): OA measures the fraction of correctly classified points over the entire dataset. In geological terms, a high OA indicates that the major lithologic units (e.g., sandstone, mudstone, conglomerate) are recognized correctly across large, laterally continuous outcrop areas, reflecting the method’s ability to honor broad stratigraphic facies.

(2) F1-score: The F1-score is the harmonic mean of precision and recall, balancing false positives (misclassifying one lithology as another) and false negatives (missing a lithology). This metric is especially important in geological contexts where thin interbeds or minor lithologic units must be detected reliably. A high F1-score demonstrates robust discrimination of these fine-scale features against the dominant stratigraphic background.

(3) Mean Intersection over Union (MIoU): MIoU calculates the average overlap between predicted and ground-truth classes on a per‐class basis. It directly quantifies boundary delineation quality, which is critical for capturing sedimentary contacts and stratigraphic interfaces. In sedimentological practice, accurately mapping these boundaries is essential for reconstructing depositional architecture and lateral continuity.

By combining OA, F1-score, and MIoU, we ensure that our evaluation not only reflects global classification success but also the method’s sensitivity to geologically significant features—sharp contacts, thin beds, and lateral continuity—thereby aligning quantitative assessment with stratigraphic principles.

## Experimental results and discussion

### Hyperparameter optimization results

**Fig. 8:**
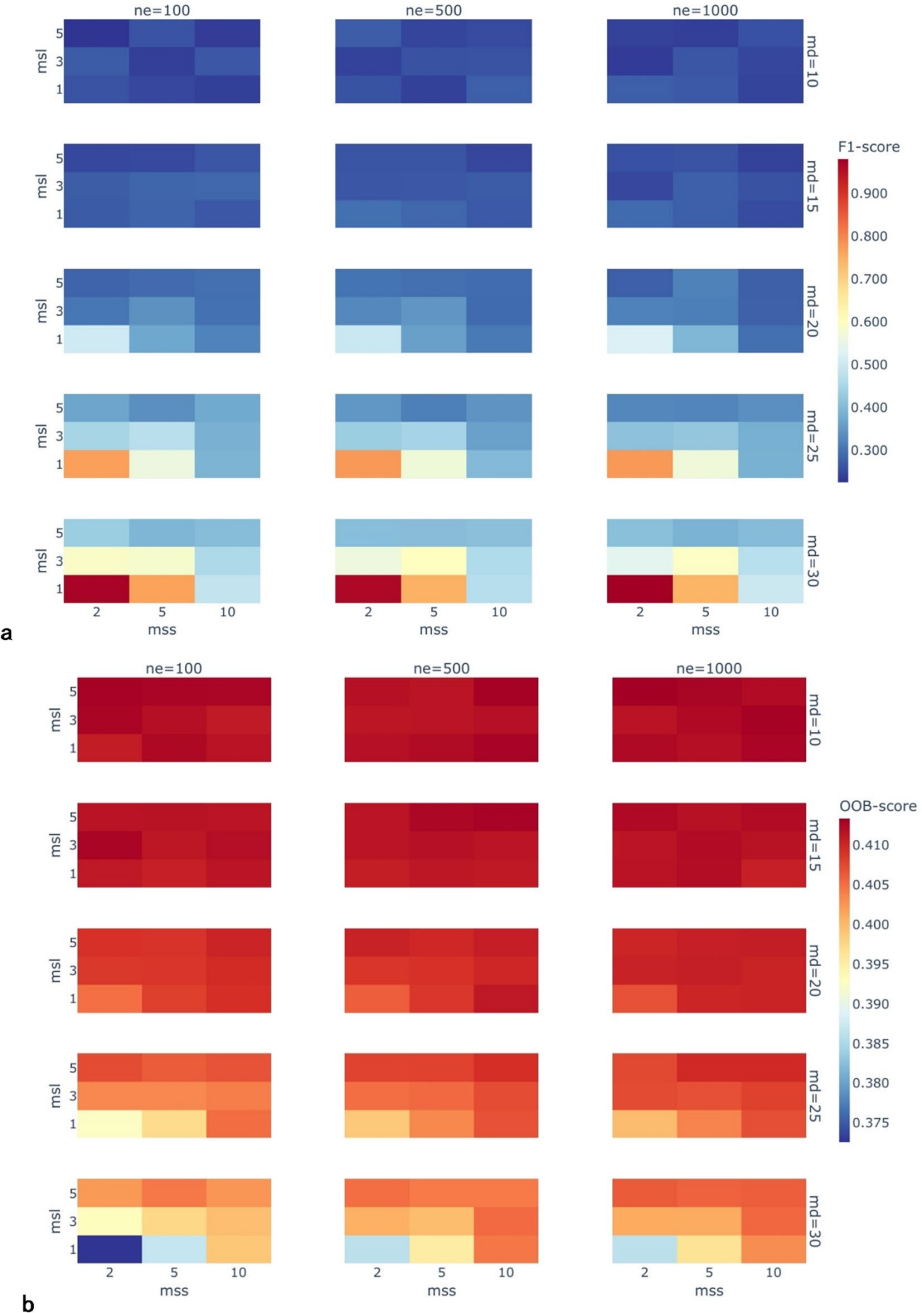
(**a**) F1-score heatmap of F1-Score Distribution across Hyperparameter Combinations. (**b**) Heatmap of Out-of-Bag (OOB) Error Distribution across Hyperparameter Combinations.

The results of the comprehensive grid search described in Sect. 2.4.3 are presented here. Figure 8a shows the weighted F1-score heatmap, and Fig. 8b shows the out-of-bag (OOB) error heatmap for all hyperparameter combinations.

As shown in the figures, the different combinations of maximum tree depth (md), number of decision trees (ne), minimum samples per leaf node (msl), and minimum samples for node splitting (mss) significantly impact the F1-score (Fig. 8a) and Out-of-Bag (OOB) error (Fig. 8b). Increasing the maximum tree depth parameter elevates the F1-score from 0.26 (md = 10) to 0.98 (md = 30) (transition from cool to warm tones in Fig. 8a), but accompanies a 5.8% decrease in the OOB score (transition from warm to cool tones in Fig. 8b). This indicates that excessively deep tree structures lead to overfitting due to heightened model complexity.

When the number of decision trees is in the low range (ne = 100→500), the F1-score improves markedly (abrupt horizontal color band shifts in Fig. 8a). In contrast, diminishing marginal effects emerge in the high range (ne = 500→1000), as the color gradient becomes smoother. When md ≥ 25, ne ≥ 500 achieves F1 > 0.75 (orange high-value cluster in Fig. 8a). Further increasing ne yields only a limited improvement of 0.02–0.05. In the potential overfitting parameter range (md ≥ 25), the F1-score at msl = 1 exceeds that at msl = 5 by over 140% (lower subplot in Fig. 8a), but mss must be ≤ 5 to maintain OOB > 0.39 (corresponding region in Fig. 8b).

Based on the spatial distribution characteristics of the geological outcrop point clouds in the study area, the optimized parameter ranges are determined as: md∈[25,30], ne∈[500,800], msl∈[1,3], mss∈[2,5]. This configuration ensures precise lithological unit boundary identification while effectively controlling model generalization errors (cool-tone regions in Fig. 8b).

### Impact of outcrop point cloud segmentation on lithology identification

#### Experimental design

To systematically evaluate the influence of geological outcrop point cloud patch segmentation on lithology identification accuracy, a dual-group ablation experiment was designed. The experimental group employs the proposed segmentation algorithm to generate topologically continuous patches, constructs patch-level feature samples by aggregating geometric feature statistics within patches, and trains a random forest model. The control group applies octree-based spatial index downsampling^41^ to the original point cloud (55 million points), retaining 6 million points, while maintaining identical classifier parameter settings. The necessity of downsampling in the control group is justified as follows: (1) Computational complexity: Direct training on the original point cloud requires processing a 55 million × 6-dimensional feature matrix (330 million dimensions), exceeding conventional computational resources, (2) Memory consumption: In a 256GB hardware environment, training the random forest model with the original point cloud occupies 240GB, with memory demands exponentially increasing during prediction; (3) Efficiency optimization: Downsampling reduces memory usage to 50% of the original data, compresses the model size to 40GB, and shortens training time from 7 h to 1 h.

#### Result analysis

**Fig. 9:**
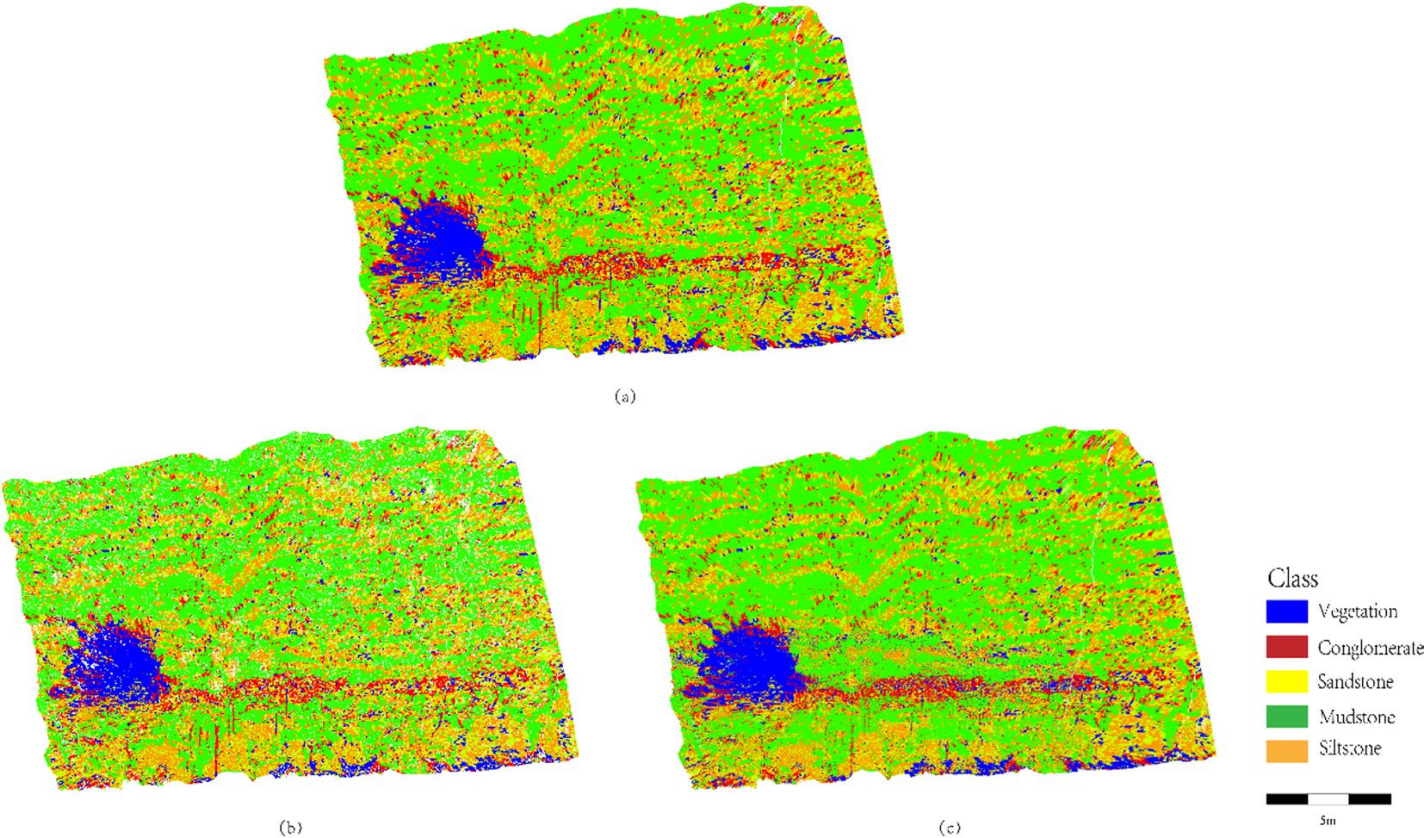
Ablation experiment results: (**a**) Ground truth labels; (**b**) SCCC (full model); (**c**) Direct Random Forest classification without SCCC.

The ablation experiment results are illustrated in Fig. 9.

A comparative analysis of lithology identification outcomes is conducted between Fig. 9b (classification after outcrop point cloud segmentation) and Fig. 9c (direct classification). In complex regions such as sandstone-mudstone thin interbeds and conglomerate-sandstone transitional zones, Fig. 9b exhibits sharp and continuous boundaries (e.g., the distinct red/yellow interface in the upper-left region). In contrast, Fig. 9c shows blurred, gradient-like transitions (e.g., the mixed yellow/green area in the central zone), attributed to local geometric noise in unsegmented point clouds misleading the classifier in transitional regions. Figure 9b achieves superior separation between vegetation (blue) and lithology zones (red/yellow/green/orange) compared to Fig. 9c. For instance, Fig. 9c displays diffuse intermixing of vegetation (blue) and mudstone (green) in the lower-right corner due to spatial confusion between vegetation and rock points in unsegmented data, whereas Fig. 9b confines vegetation to isolated blocks via segmentation preprocessing, demonstrating high robustness against non-lithological interference. In millimeter-scale sandstone-mudstone interbedded structures (central yellow-green banded region in Fig. 9b), the alternating lithological layers are fully preserved, while Fig. 9c exhibits diffusion of mudstone (green) into sandstone (yellow), caused by oversensitivity of direct classification to local point density variations. This confirms that the segmentation strategy enhances discrimination of microscale lithological structures through stratigraphic unit aggregation.

**Fig. 10:**
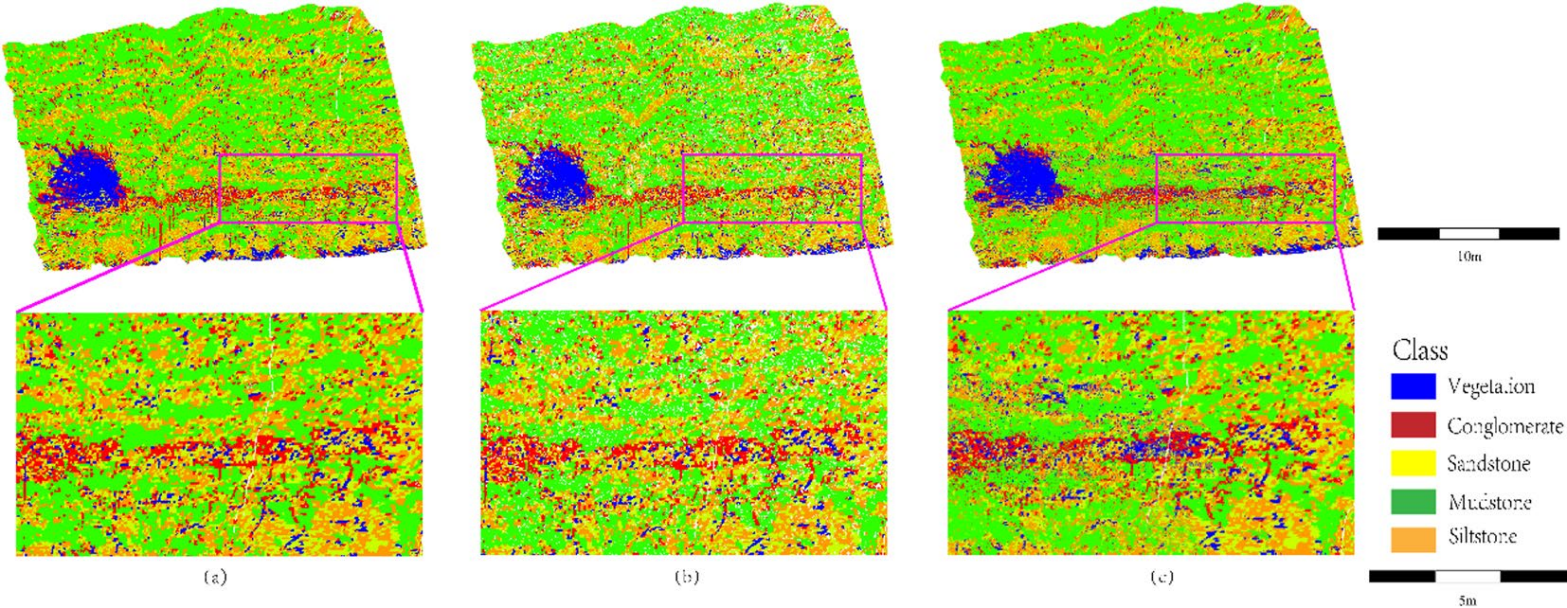
Comparison of lithology transition zone boundary identification (highlighting sharpness differences in sandstone-mudstone thin interbeds and conglomerate-sandstone transitions between SCCC and direct classification).

**Fig. 11:**
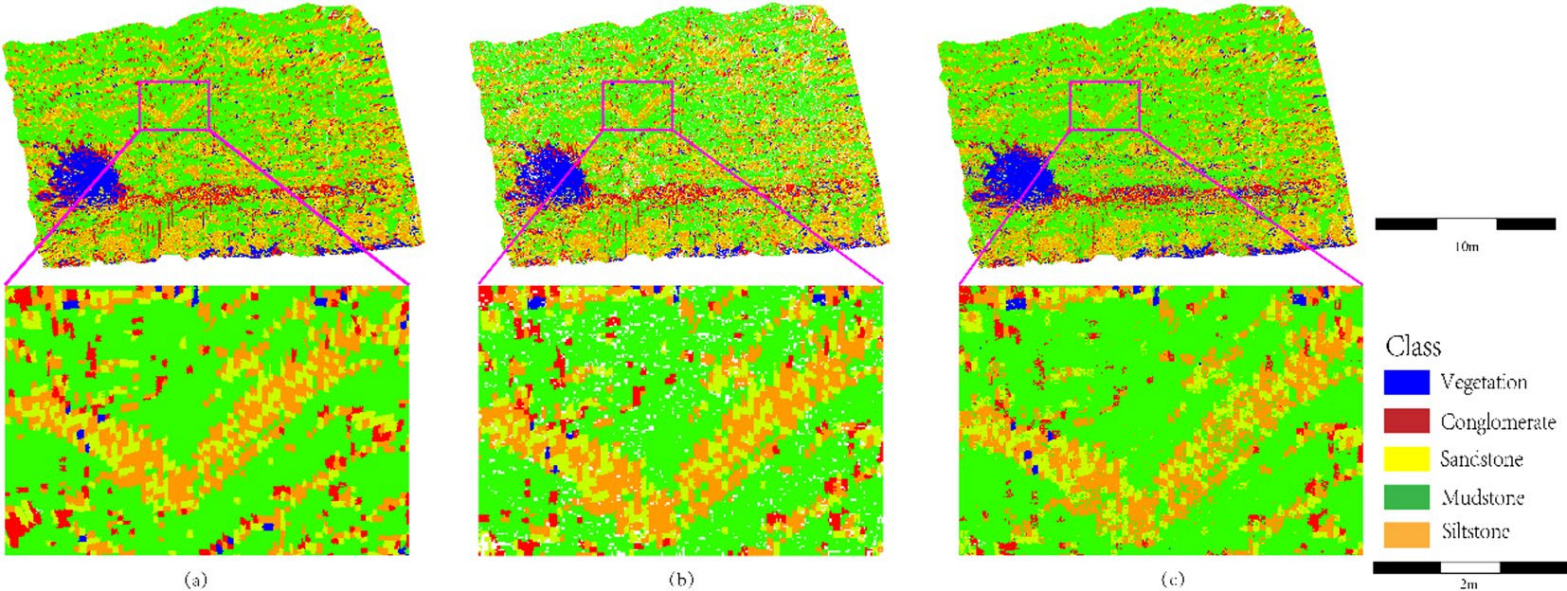
Boundary characteristics of complex lithology contact zones (comparing geometric preservation and noise suppression between SCCC and direct classification).

As shown in Fig. 10, the boundary sharpness of sandstone-mudstone thin interbeds and conglomerate-sandstone transitional zones is compared between the SCCC method and direct classification. In the pink box-marked conglomerate (red)-sandstone (yellow) transitional zone (Fig. 10b), the contact surface exhibits a clear linear boundary (sharp red/yellow demarcation), preserving the high-curvature morphological characteristics of conglomerate clast edges (see zoomed area in the lower left of Fig. 10b), which aligns with the physical properties of coarse conglomerate accumulations. In contrast, the same region in Fig. 10c displays sandstone (yellow) intrusion into conglomerate (red) (see zoomed area in the lower right of Fig. 10c), resulting in blurred boundaries due to geometric noise interference from unsegmented point clouds. In the sandstone-mudstone thin interbedded extension zone (yellow-green alternating bands on the right side of the pink box), Fig. 11b resolves the thin-layered structures into discrete strip-shaped units (alternating yellow sandstone and green mudstone), consistent with field-measured lamellar sedimentary sequences. Conversely, Fig. 10c exhibits mudstone (green) misclassification into sandstone (yellow) (see lower-right zoomed area), with partial loss of thin-layer details, indicating insufficient sensitivity of direct classification to microscale lithological alternations. Black speckle noise in Fig. 10c is attributed to the absence of patch-level feature aggregation and fixed thresholds failing to distinguish true mudstone surface points from noise. The unsegmented point cloud in Fig. 10c lacks spatial continuity constraints, further validating the superiority of stratigraphically constrained clustering in lithology identification.

As illustrated in Fig. 11, the geometric morphology preservation and noise suppression effects of the stratigraphically constrained clustering strategy are compared in complex lithology contact zones, such as sandstone/siltstone-mudstone interbedded layers and conglomerate-sandstone transitional boundaries. In the purple box-marked sandstone (yellow)/siltstone (orange)-mudstone (green) interbedded region (Fig. 11b), alternating sandstone (yellow) and mudstone (green) layers exhibit sharp, continuous linear boundaries with uniform thickness, consistent with field-measured thin interbedded sedimentary characteristics. The segmentation strategy effectively captures millimeter-scale bedding structures through spatial continuity constraints on stratigraphic units. In contrast, Fig. 11c shows blurred boundaries in the same region, with mudstone (green) diffusing into sandstone (yellow), caused by local density fluctuations in unsegmented point clouds misleading the classifier. Above the purple box, the conglomerate (red)-sandstone (yellow) contact zone in Fig. 11b retains high-curvature edges of conglomerate clasts, with no noise infiltration in sandstone areas. Conversely, Fig. 11c exhibits clusters of black speckle noise at conglomerate edges and false sandstone (yellow) intrusions into conglomerate (red). These artifacts arise from outlier points caused by rough conglomerate surfaces (e.g., multi-return laser reflections) and geometric discontinuities (e.g., interstitial voids between clasts) in unsegmented data. For the sandstone (yellow)-siltstone (orange) transition in Fig. 11b, gradual yet distinct layering aligns with sedimentological gradational principles, whereas Fig. 11c displays sporadic siltstone (orange) distribution in high-energy sandstone zones (e.g., alluvial fan margins), violating depositional dynamics. This confirms that hierarchical clustering with stratigraphic continuity constraints mitigates lithological misclassification caused by local point cloud anomalies.

**Fig. 12:**
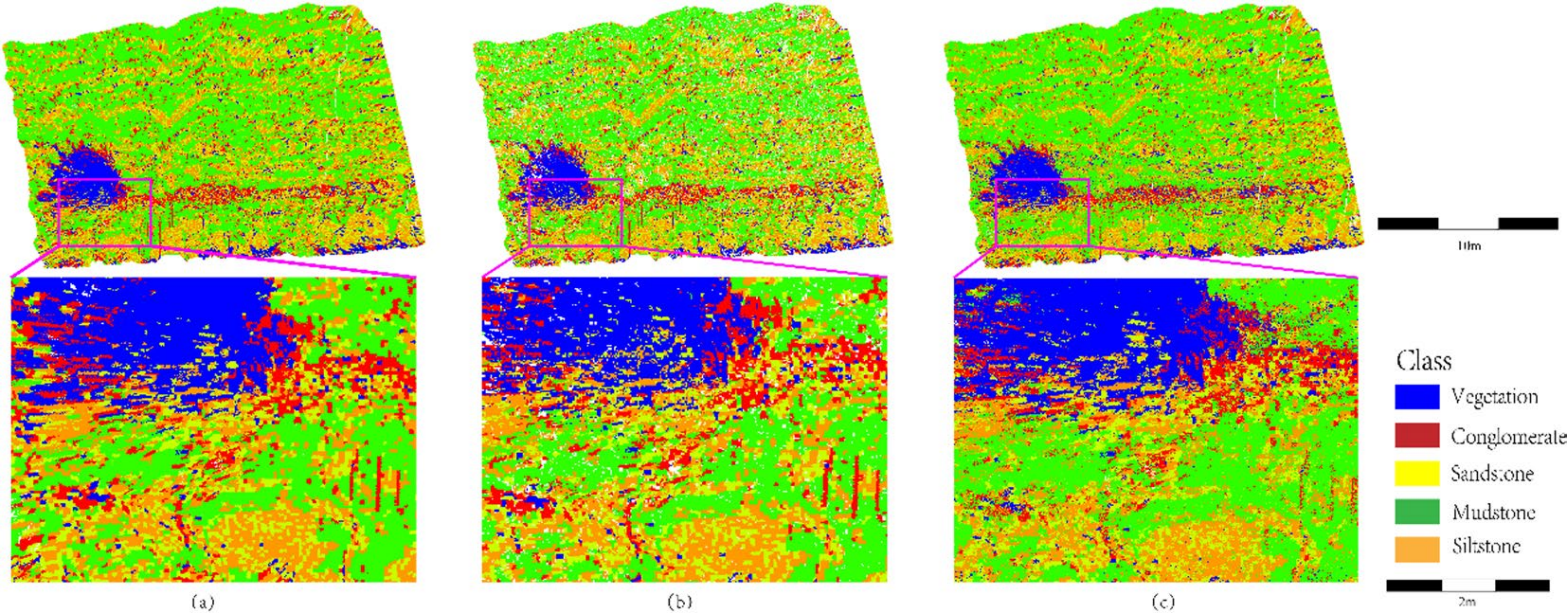
Multi-lithology spatial distribution and vegetation isolation effects (highlighting lithological discrimination robustness and environmental adaptability of SCCC in vegetation-proximal zones).

As shown in Fig. 12, the spatial distribution of multiple lithologies and vegetation isolation effects are compared between the stratigraphically constrained clustering strategy and direct classification. In regions with complex lithological combinations (Fig. 12b), vegetation (blue) is strictly confined to fractures or outcrop edges, separated from mudstone (green) by physical intervals, consistent with the ecological principle of root systems preferentially colonizing fractured zones. In contrast, Fig. 12c exhibits mixed blue-green patches where vegetation and mudstone overlap on slopes, caused by coordinate offsets from vegetation sway in unsegmented point clouds, leading to misclassification of mudstone near fractures as vegetation. In the conglomerate (red)-sandstone (yellow) contact zone, the SCCC method preserves the high-curvature morphology of conglomerate clast accumulation (Fig. 12b), while sandstone regions remain noise-free. Conversely, Fig. 12c displays a red-yellow gradient zone with black speckles, indicating sandstone-conglomerate confusion and false boundaries. The unsegmented data in Fig. 12c fail to suppress surface roughness-induced noise (e.g., laser multi-return outliers) or geometric discontinuities (e.g., clast gaps). Additionally, in the thin interbedded zone (yellow in Fig. 12b), sandstone maintains uniform classification due to segmentation-based density anomaly filtering, whereas Fig. 12c shows scattered yellow-orange-green noise from microcrack sensitivity in direct classification, disrupting sedimentary coherence. These results validate that the proposed method enhances lithological sensitivity and isolates vegetation interference through spatial continuity constraints.

The values reported in Table 5 were produced by a controlled ablation experiment comparing two pipelines applied to the same outcrop dataset. In the experimental pipeline (SCCC, including its integrated patch-level aggregation module), the preprocessed point cloud was first segmented into patch-level units using the stratigraphically constrained clustering; patch features were then aggregated by SCCC’s internal patch-level aggregation module to form ≈ 36,000 samples. In the control pipeline (direct classification), the original point cloud was octree-down sampled to retain ~ 6 million points and Random Forest classification was applied at the point level. For both pipelines we used stratified sampling to split samples into training and test sets (95% training / 5% testing) to preserve class balance; Random Forest hyperparameters were selected by the grid search procedure described in Sect. 2.3.3. For the experimental group, predicted patch labels were inversely mapped to the original points via the patch index (full-scene inversion) so that evaluation metrics (Overall Accuracy, weighted F1-score, and Mean IoU) were computed at the point level and are therefore directly comparable between pipelines. All reported metrics are percentages. The GitHub repository linked in the Data Availability section contains the code and exact parameter settings used for reproducibility.

**Table 5:** Ablation experiment evaluation metrics.

Evaluation Metric	Proposed Method (%)	Baseline (%)	Improvement (Δ%)
Overall Accuracy	94.64	90.98	4.02%
F1-score	94.58	90.88	4.07%
Mean IoU	90.87	86.35	5.23%

Quantitative comparisons between the experimental and control groups are summarized in Table 5. The proposed stratigraphically constrained clustering strategy (experimental group) significantly outperforms direct classification (control group) in lithology identification metrics. The overall accuracy (OA) increases by 4.02% (94.64% vs. 90.98%), F1-score improves by 4.07% (94.58% vs. 90.88%), and mean intersection over union (MIoU) rises by 5.23% (90.87% vs. 86.35%). These enhancements are attributed to the dynamic density threshold algorithm, which merges fragmented clusters in transition zones (e.g., sandstone-mudstone thin interbeds) through density continuity criteria (Eq. 4), reducing misclassification rates. The adjacency-based cluster merging algorithm (Fig. 7) effectively filters non-lithological interference (e.g., vegetation sway points, conglomerate surface outliers). The experimental group processes only 10,000 patch samples (vs. 6 million points in the control group), reducing memory consumption by 83.3% (40 GB vs. 240 GB) and training time by 85.7% (1 h vs. 7 h). These results demonstrate that the stratigraphic continuity-guided segmentation strategy achieves a balance between computational efficiency and lithological boundary precision.

### Comparative experimental results and analysis

**Fig. 13:**
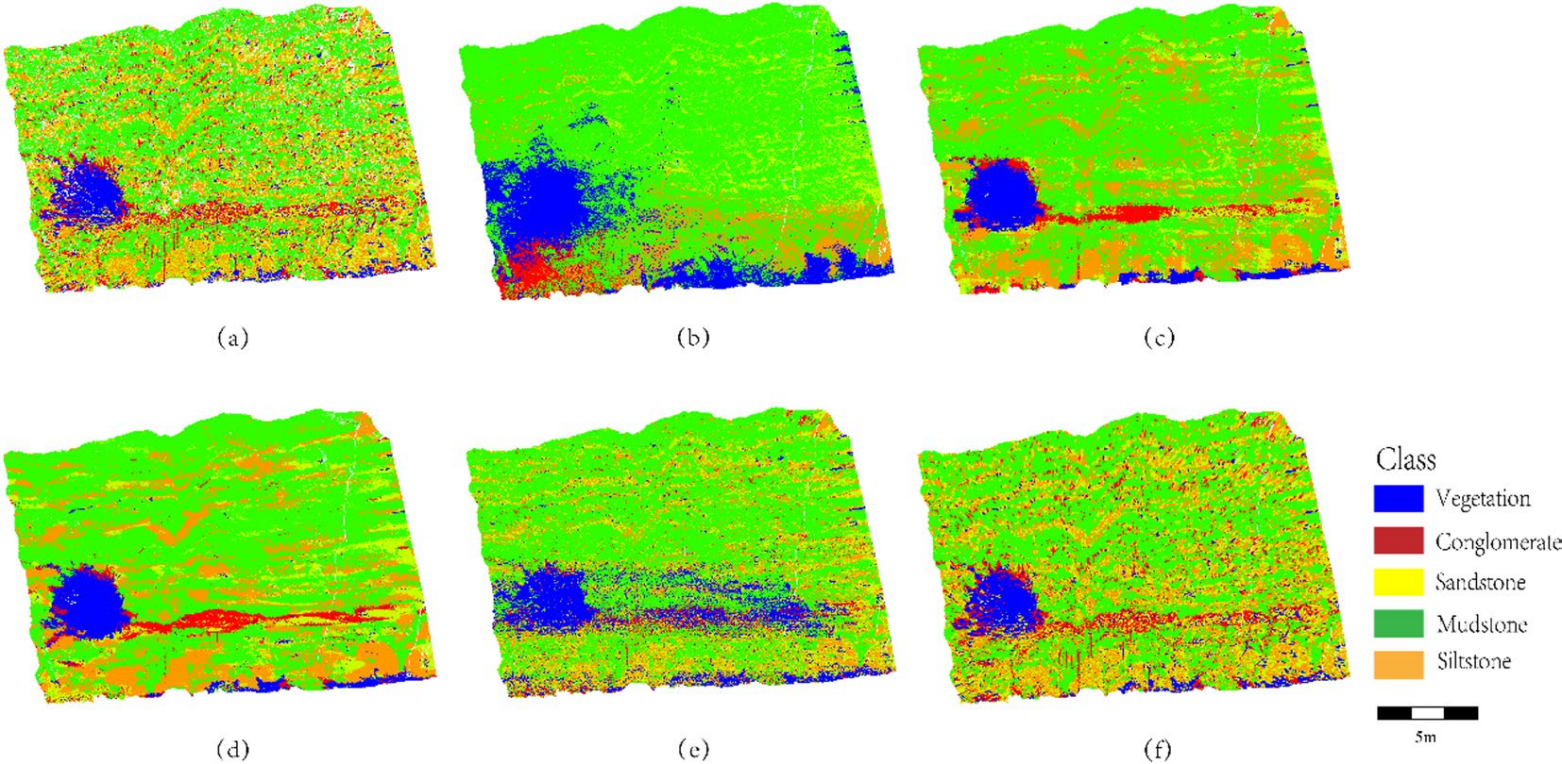
Comparative results of lithology identification methods (**a-e**: SCCC-RF, SVM, XGBoost, PointNet, K-NN; **f**: Ground truth).

The effectiveness of the proposed stratigraphic clustering method is validated through comparative experiments with baseline models, including SVM, XGBoost, PointNet, and K-NN. The results are visualized in Fig. 13.

As shown in Fig. 13a (SCCC-RF), the proposed method achieves the highest boundary precision in complex geological scenarios. For instance, sandstone-mudstone contact zones exhibit linear, sharp boundaries (e.g., straight yellow-green interfaces in Fig. 13a), with reduced edge jaggedness compared to PointNet. The global constraints from geometric covariance matrices (Eq. 5) preserve the macroscopic strike of thin sandstone layers. Traditional machine learning methods (SVM/XGBoost, Fig. 13b-c) show diffuse confusion in conglomerate-sandstone transition zones (red-yellow overlaps), attributed to insufficient nonlinear mapping capabilities. PointNet (Fig. 13d) introduces “ink diffusion” artifacts (green mudstone regions with burr-like boundaries), as its point-level feature pooling ignores local stratigraphic continuity. K-NN (Fig. 13e) generates sporadic noise in siltstone-mudstone zones (orange-green speckling), failing to maintain spatial coherence due to its local sensitivity. These results confirm that the integration of stratigraphic continuity constraints and multimodal features in SCCC-RF significantly enhances boundary integrity and lithological discrimination robustness.

**Fig. 14:**
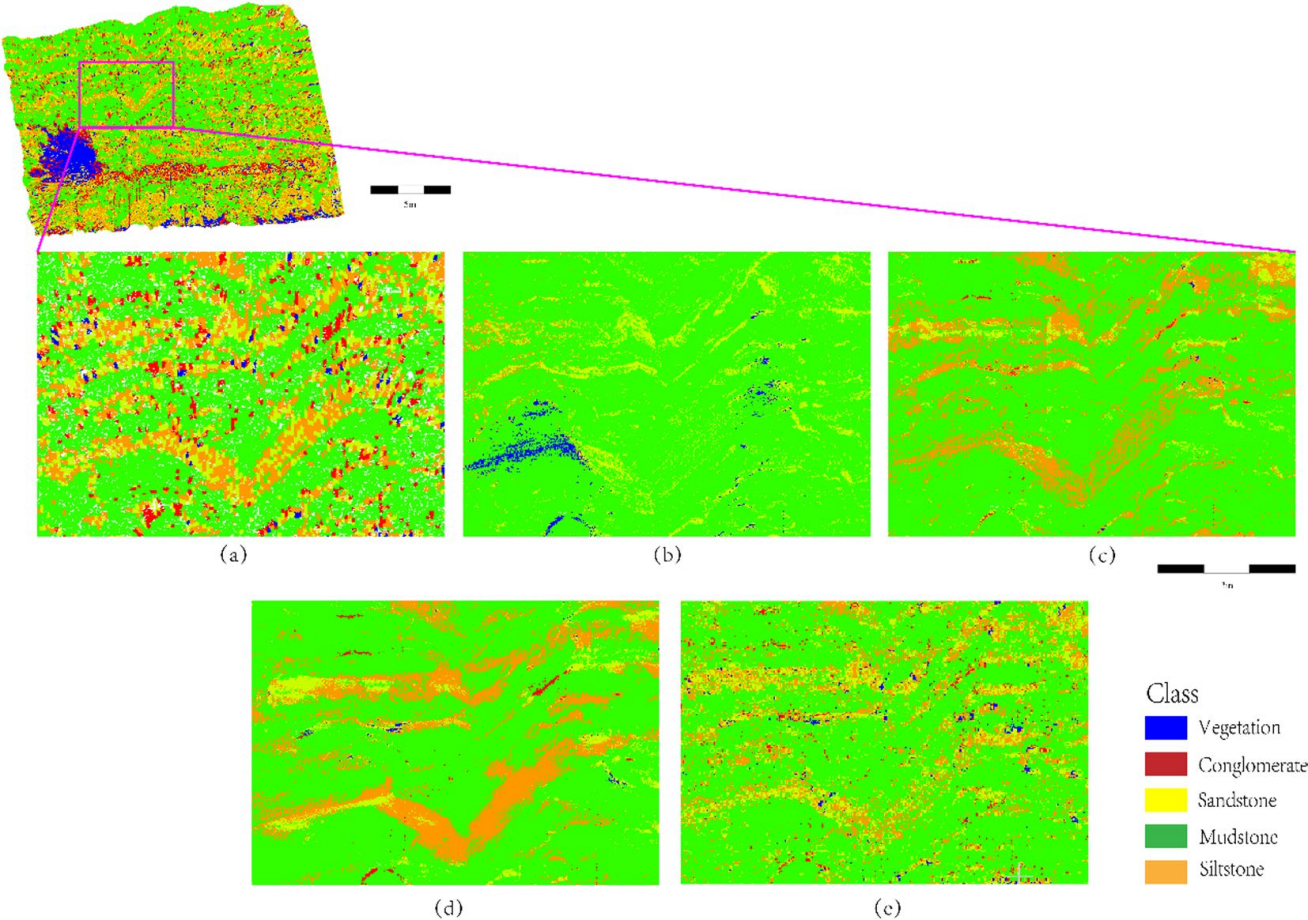
Lithology identification in thin-bedded sandstone-siltstone-mudstone interlayers (highlighting boundary precision and noise suppression of SCCC-RF compared to baseline methods).

As illustrated in Fig. 14, the lithology identification performance of the SCCC-RF method is compared with SVM, XGBoost, PointNet, and K-NN in thin-bedded sandstone-siltstone-mudstone interlayers. In Fig. 14a (SCCC-RF), the alternating thin layers of sandstone (yellow) and mudstone (green) exhibit sharp, continuous boundaries with uniform thickness, accurately reflecting the lamellar sedimentary sequences observed in field measurements. The SCCC method effectively suppresses local point cloud noise through stratigraphic continuity constraints and enhances the discrimination of fine-grained lithologies (e.g., siltstone vs. mudstone) via multimodal feature fusion. In contrast, SVM (Fig. 14b) and XGBoost (Fig. 14c) produce blurred boundaries (e.g., mudstone diffusion into sandstone) and non-geological grid-like artifacts due to limited nonlinear modeling capabilities and oversensitivity to local density fluctuations. PointNet (Fig. 14d), while capturing global features, introduces burr-like boundaries in mudstone regions (green) caused by ignorance of spatial continuity, leading to lithological confusion at thin-layer edges. K-NN (Fig. 14e) generates scattered speckle noise (orange siltstone mixed with green mudstone), failing to maintain spatial coherence due to its isolated decision mechanism. These results demonstrate that the SCCC-RF method achieves superior accuracy in resolving microscale sedimentary structures while ensuring geological plausibility.

**Fig. 15:**
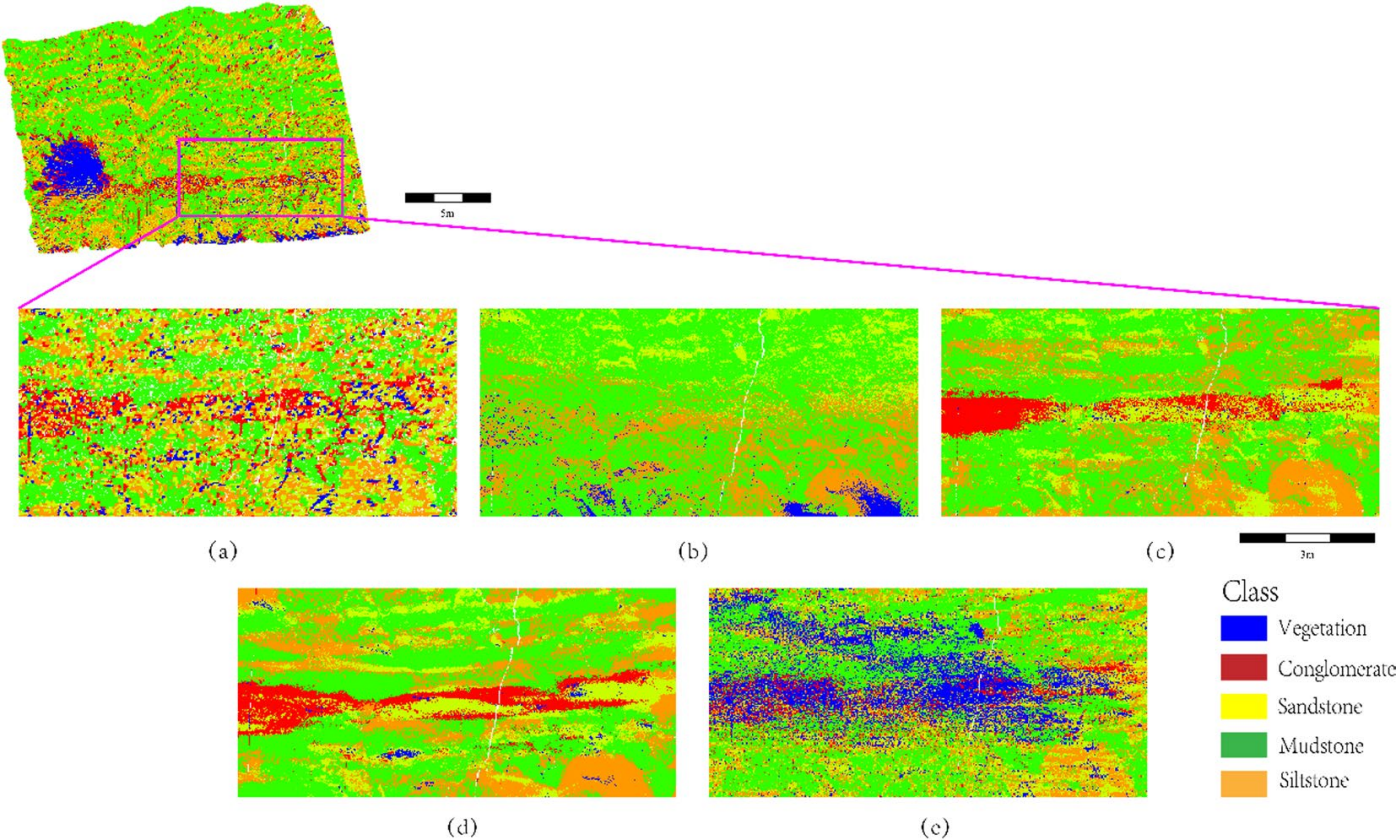
Lithology identification in conglomerate-dominated formations (highlighting depositional plausibility and noise robustness of SCCC-RF compared to traditional methods).

As shown in Fig. 15, the lithology identification performance of the SCCC-RF method is evaluated in conglomerate-dominated formations and compared with baseline models (SVM, XGBoost, PointNet, K-NN). In Fig. 15a (SCCC-RF), conglomerates (red) are accurately concentrated in high-energy depositional units (e.g., alluvial fan margins), retaining the high-curvature morphology of coarse clast accumulation. Vegetation (blue) and mudstone (green) are strictly separated without diffuse intermixing, and sandstone regions (yellow) remain noise-free. The SCCC method effectively suppresses outlier interference caused by conglomerate surface roughness through dynamic density threshold constraints and enhances sandstone-conglomerate differentiation by integrating spectral distribution entropy features (validated via XRD analysis). In contrast, SVM (Fig. 15b) misclassifies iron-bearing siltstone (with a 2240 nm absorption peak) as conglomerate due to reliance on single-spectral features. XGBoost (Fig. 15c) exhibits overfitting-induced overdetection of conglomerate (red overspreading), violating spatial depositional facies patterns. PointNet (Fig. 15d) and K-NN (Fig. 15e) generate black speckle clusters at conglomerate edges and false sandstone intrusions (yellow-red gradients), attributed to unsegmented point cloud geometric discontinuities (e.g., clast gaps) and sensitivity to surface roughness. These results demonstrate that SCCC-RF achieves geologically consistent lithology mapping in conglomerate-rich environments while balancing precision and interpretability.

**Fig. 16:**
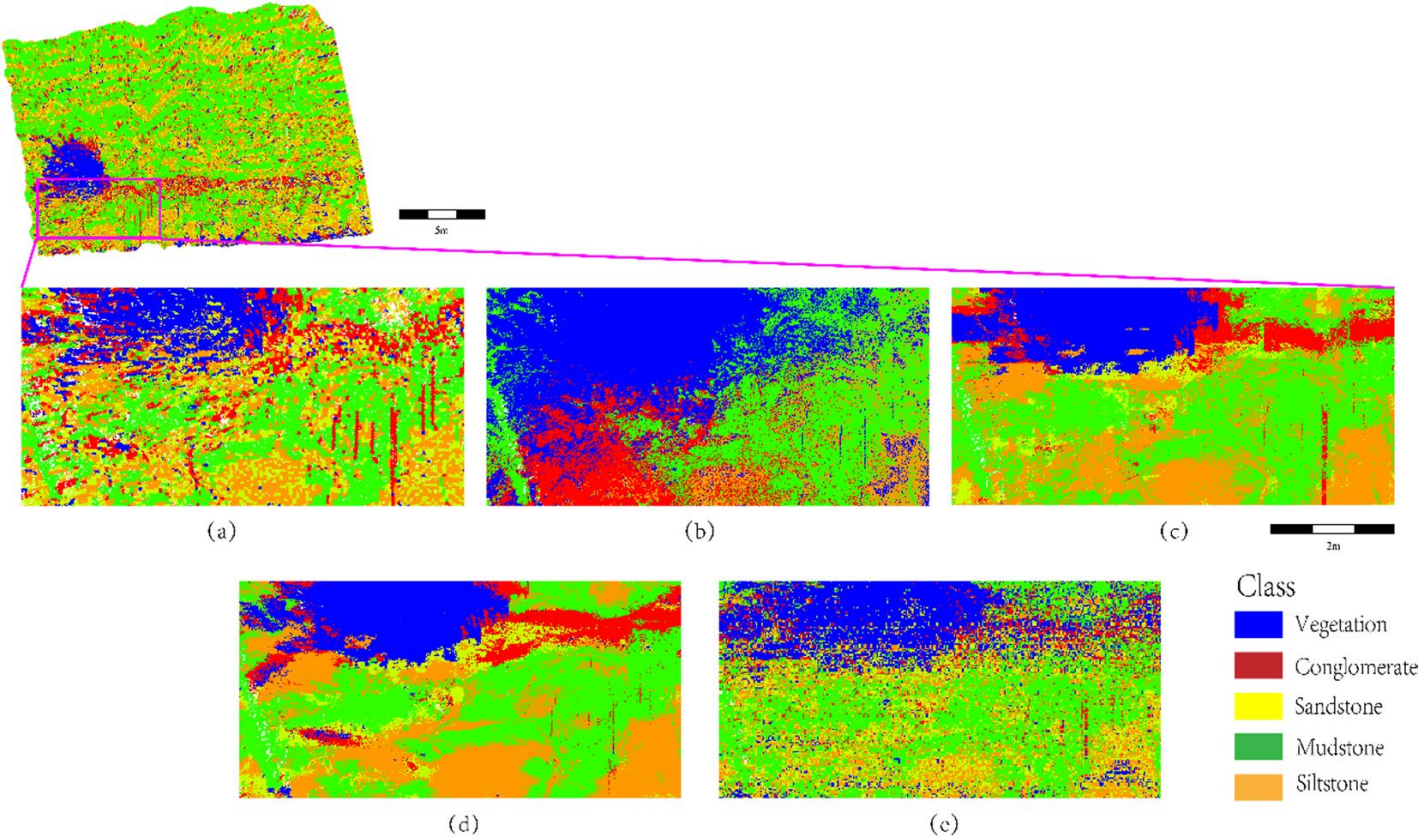
Comparative analysis of lithology identification in vegetation proximity (highlighting SCCC-RF’s robustness in isolating vegetation interference and maintaining geological plausibility).

As illustrated in Fig. 16, the lithology identification performance of the SCCC-RF method is evaluated in vegetation-proximal regions and compared with baseline models (SVM, XGBoost, PointNet, K-NN). In Fig. 16a (SCCC-RF), vegetation (blue) is strictly confined to fractures or outcrop edges, separated from mudstone (green) by physical intervals, aligning with the ecological principle of root systems preferentially colonizing fractured zones. The high-curvature morphology of conglomerate clast edges (red) and noise-free sandstone regions (yellow) are preserved, demonstrating effective suppression of surface roughness-induced classification noise through stratigraphic continuity constraints. In contrast, SVM (Fig. 16b) and XGBoost (Fig. 16c) misclassify mudstone as vegetation (blue-green mixed patches on slopes) due to spatial confusion in unsegmented point clouds. PointNet (Fig. 16d) allows sandstone (yellow) to infiltrate vegetation zones (blue) due to global feature pooling neglecting local contextual constraints. K-NN (Fig. 16e) produces scattered yellow-orange-green noise near fractures, disrupting sedimentary coherence via its isolated point-based decisions. These results validate the environmental adaptability and robustness of SCCC-RF in vegetation-interfered geological scenarios, achieving precise lithology-vegetation separation while preserving depositional integrity.

**Table 6:** Comparative experiment evaluation metrics.

Method	F1-score (%)	OA (%)	MIoU (%)
SCCC-RF	94.58	94.64	90.87
SVM	45.61	53.85	26.33
XGBoost	60.74	63.94	40.92
PointNet	68.36	65.43	53.54
K-NN	51.40	54.15	30.90

The quantitative evaluation metrics of the five methods (SCCC-RF, SVM, XGBoost, PointNet, K-NN) are compared in Table 6. The SCCC-RF method achieves a significant lead across all core metrics: F1-score (94.58%, 26.22% higher than the suboptimal method PointNet), overall accuracy (OA: 94.64%, 30.7% higher than XGBoost), and mean intersection over union (MIoU: 90.87%, 37.33% higher than PointNet). These results confirm the superiority of the stratigraphic continuity-guided clustering strategy in classification accuracy, boundary integrity, and semantic coherence. The SCCC-RF method effectively resolves challenges such as vegetation interference, conglomerate-sandstone differentiation, and thin interbedded structure preservation, providing a robust framework for intelligent geological interpretation.

## Discussion

### Method comparison and evaluation

The proposed Stratigraphically Constrained Continuous Clustering (SCCC) method demonstrates innovation by deeply integrating geological principles with computational models, offering a new technical pathway for high-precision and interpretable lithology identification. The quantitative encoding of stratigraphic continuity constraints into clustering criteria (e.g., dynamic density thresholds and adjacency-based merging) significantly reduces missegmentation rates in transitional zones (e.g., sandstone-mudstone interbeds) compared to traditional density clustering methods like DBSCAN. Furthermore, the patch-level aggregation module integrated within SCCC enhances fine-grained lithology discrimination (e.g., siltstone vs. mudstone) through multimodal fusion of geometric covariance matrices and spectral entropy, outperforming single-modality approaches such as XGBoost^42,43^.

Computational efficiency is optimized via lightweight hash table-based label mapping, reducing memory consumption by 83.3% (40 GB vs. 240 GB) and training time by 85.7% (1 h vs. 7 h) compared to point-level methods. The hierarchical clustering strategy enables geological-unit parallelization, demonstrating potential for real-time field interpretation.

However, SCCC still exhibits limitations in handling gradual lithological transitions (e.g., alluvial sand–silt gradations), which may require more adaptive segmentation strategies beyond global scale tuning. Although the sensitivity analysis identified an optimal fixed scale for this dataset, dynamically adjusting scale at local levels could better accommodate transitional lithologies. Moreover, the exclusion of hyperspectral mineralogical features restricts differentiation between conglomerates and weathered sandstones. In addition, the assumption of stratigraphic lateral continuity may not strictly hold in geological contexts dominated by syn-sedimentary tectonics or erosional truncations, where strata can be discontinuous or highly deformed. In such cases, rigid stratigraphic constraints may oversimplify or misrepresent local lithological variability, suggesting the need for adaptive stratigraphic constraints or integration with structural geological information.

Despite these limitations, a key methodological advance of SCCC lies in its explicit encoding of stratigraphic inheritance principles into the clustering process. By incorporating Steno’s Law of Original Horizontality and the Principle of Lateral Continuity into hierarchical clustering criteria, the method ensures that the resulting patches are not only geometrically coherent but also geologically plausible. This geologically informed constraint bridges the gap between purely data-driven clustering and sedimentological interpretation, enhancing the interpretability and robustness of automated lithology identification in complex outcrop settings.

### Influence of scale parameter

To evaluate the effect of segmentation granularity on lithology classification performance, we conducted a scale sensitivity analysis. Eleven different scale parameters ranging from 5 to 100 were tested, incremented by 10 (except for the first two values). These parameters were applied during the neighborhood density-based clustering step, where they control the cutoff distance d_c_ that defines the initial patch granularity of stratigraphic units.

**Fig. 17:**
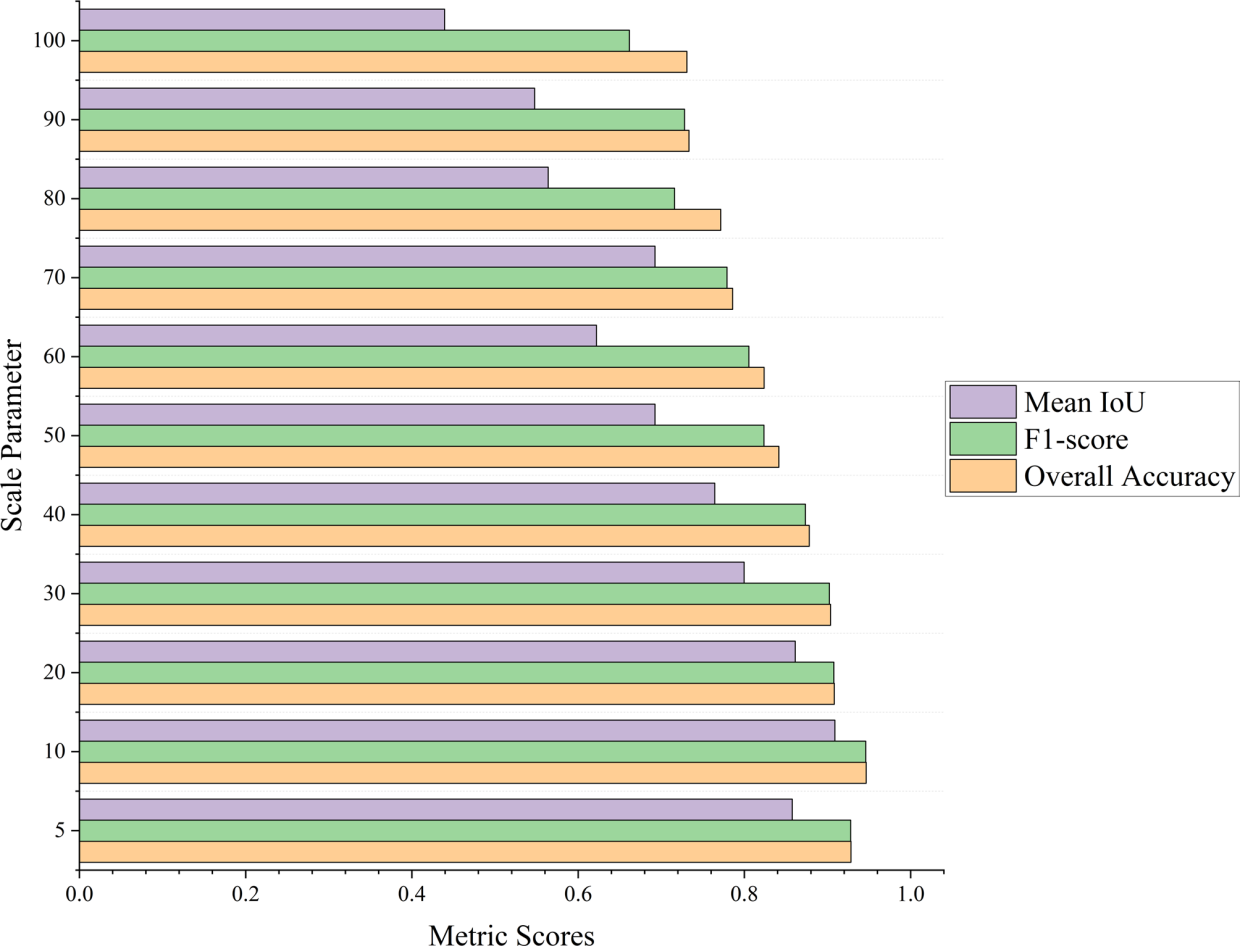
Classification performance under different scale parameter settings based on Overall Accuracy (OA), F1-score, and mean Intersection over Union (MIoU).

The classification performance under each scale setting was evaluated using three standard metrics: Overall Accuracy (OA), F1-score, and mean Intersection over Union (MIoU). The results are shown in Fig. 17.

As illustrated in Fig. 17, classification performance exhibits a distinct trend with varying scale. At very small scales (e.g., 5), the segmentation is overly fine-grained, leading to noisy patch boundaries and fragmented units, which results in reduced classification robustness despite relatively high OA and F1 scores. As the scale increases, performance improves, reaching a peak at scale = 10, where all three metrics (OA = 0.9464, F1 = 0.9458, MIoU = 0.9087) attain their highest values. This suggests that moderate patch granularity preserves the internal consistency of lithologic units while still capturing meaningful boundaries.

Beyond scale = 10, further increases lead to coarse segmentation, which tends to merge geologically distinct units. As a result, all three metrics show a gradual decline. Notably, MIoU drops sharply from 0.861 at scale = 20 to 0.4393 at scale = 100, indicating that coarse segmentation severely impairs boundary-level accuracy. This trend highlights the importance of maintaining an optimal balance between detail and generalization in stratigraphic partitioning. Based on this analysis, we selected scale = 10 as the optimal setting in our pipeline. This choice ensures sufficient structural detail while avoiding over fragmentation or semantic ambiguity. The experiment also reinforces the robustness and adaptability of our clustering framework in capturing meaningful stratigraphic continuity across different scales.

## Conclusion

To address the challenges of boundary ambiguity and classification accuracy degradation in lithology identification of outcrop point clouds under complex geological scenarios, this study proposes a Stratigraphically Constrained Continuous Clustering method (SCCC). The method integrates a density-adaptive point cloud clustering algorithm that encodes stratigraphic continuity principles, significantly reducing missegmentation rates in sandstone-mudstone transitional zones. A patch-level aggregation module integrated within the SCCC framework combines geometric covariance matrices and spectral distribution entropy to enhance the separability between sandstone and conglomerate. A hash table-based label mapping system achieves full-scene inversion of 50 million point cloud labels, optimizing computational efficiency. Experimental validation on the Qingshuihe Formation outcrop dataset in the Junggar Basin demonstrates that SCCC outperforms traditional machine learning (SVM, XGBoost) and deep learning methods (PointNet) in accuracy metrics (OA: 94.64%, F1-score: 94.58%, MIoU: 90.87%), particularly improving IoU by 31.5% in thin interbedded boundaries and reducing salt-and-pepper noise. The method achieves high precision (IoU = 92.1%) in identifying mudstone weak interlayers, enabling early warnings for high-risk landslide zones. SCCC is applicable to mineral deposit identification and structural analysis, advancing intelligent geological exploration. Future research should develop adaptive density threshold algorithms to enhance segmentation accuracy for gradual lithologies such as alluvial deposits. Bimodal feature aggregation could integrate hyperspectral and geochemical data to further improve lithology discrimination^44^.

## Data Availability

The core implementation of the proposed Stratigraphy-Guided Continuous Clustering with Random Forest (SGCC-RF) method is publicly available on GitHub to facilitate reproducibility and further research. The repository contains well-documented Python and C++ code corresponding to the key modules described in this paper: 1) Stratigraphy-Constrained Hierarchical Clustering (C++): This module implements the core geological constraint logic. 2) Patch-level Feature Aggregation Operator (PFAO) & Sample Generation (C++). 3) Random Forest Classification & Hyperparameter Optimization (Python). 4) Hash Table-Based Label Mapping (Python)GitHub Repository: https://github.com/GBQ134/SGCC.
